# Treatment regimen determines whether an HIF-1 inhibitor enhances or inhibits the effect of radiation therapy

**DOI:** 10.1038/sj.bjc.6604939

**Published:** 2009-02-17

**Authors:** H Harada, S Itasaka, Y Zhu, L Zeng, X Xie, A Morinibu, K Shinomiya, M Hiraoka

**Affiliations:** 1 Department of Radiation Oncology and Image-applied Therapy, Kyoto University Graduate School of Medicine, 54 Shogoin Kawahara-cho, Sakyo-ku, Kyoto 606-8507, Japan; 2 Nano-medicine Merger Education Unit, Kyoto University, 54 Shogoin Kawahara-cho, Sakyo-ku, Kyoto 606-8507, Japan; 3 Department of Radiation Medicine, Fourth Military Medical University, 17 Chargle West Road, Xi’an, Shaanxi 710032, China

**Keywords:** radiation therapy, tumour hypoxia, hypoxia-inducible factor-1, molecular imaging

## Abstract

Hypoxia-inducible factor-1 (HIF-1) has been reported to promote tumour radioresistance; therefore, it is recognised as an excellent target during radiation therapy. However, the inhibition of HIF-1 in unsuitable timing can suppress rather than enhance the effect of radiation therapy because its anti-angiogenic effect increases the radioresistant hypoxic fraction. In this study, we imaged changes of HIF-1 activity after treatment with radiation and/or an HIF-1 inhibitor, YC-1, and optimised their combination. Hypoxic tumour cells were reoxygenated 6 h postirradiation, leading to von Hippel-Lindau (VHL)-dependent proteolysis of HIF-1*α* and a resultant decrease in HIF-1 activity. The activity then increased as HIF-1*α* accumulated in the reoxygenated regions 24 h postirradiation. Meanwhile, YC-1 temporarily but significantly suppressed HIF-1 activity, leading to a decrease in microvessel density and an increase in tumour hypoxia. On treatment with YC-1 and then radiation, the YC-1-mediated increase in tumour hypoxia suppressed the effect of radiation therapy, whereas on treatment in the reverse order, YC-1 suppressed the postirradiation upregulation of HIF-1 activity and consequently delayed tumour growth. These results indicate that treatment regimen determines whether an HIF-1 inhibitor enhances or inhibits the therapeutic effect of radiation, and the suppression of the postirradiation upregulation of HIF-1 activity is important for the best therapeutic benefit.

A tumour-specific microenvironment, hypoxia, is associated with resistance to radiation therapy because the depletion of oxygen disturbs radiolysis of H_2_O and reduces the production of reactive and cytotoxic species, and because radiation-induced DNA damage is fixed and irrepairable under normoxia (Thomlinson and Gray, 1955; Brown and Wilson, 2004). In addition to these chemical mechanisms, a biological mechanism mediated by hypoxia-inducible factor-1 (HIF-1) has been reported to play an important role in hypoxia-related radioresistance (Moeller and Dewhirst, 2006).

Hypoxia-inducible factor-1 is a heterodimeric transcription factor composed of an *α*-subunit (HIF-1*α*) and a *β*-subunit (HIF-1*β*), and its activity is mainly dependent on the stability and modification of the former (Wang *et al*, 1995). Under normoxic conditions, prolyl hydroxylation and subsequent ubiquitination of the oxygen-dependent degradation (ODD) domain of HIF-1*α* leads to a rapid degradation of HIF-1*α* protein with a half-life of 5–8 min (Berra *et al*, 2001; Jaakkola *et al*, 2001). On the other hand, HIF-1*α* is stabilised and interacts with HIF-1*β* under hypoxic conditions (Wang *et al*, 1995). The resultant heterodimer, HIF-1, binds to its cognate DNA sequence, the hypoxia-responsive element (HRE), and induces the expression of various factors such as vascular endothelial cell growth factor (VEGF; Norris and Millhorn, 1995; Forsythe *et al*, 1996; Semenza, 2001). Vascular endothelial cell growth factor has been reported to not only induce angiogenesis but also protect endothelial cells from the cytotoxic effects of irradiation and consequently increase tumour radioresistance (Gorski *et al*, 1999; Moeller *et al*, 2004; Zeng *et al*, 2008). Therefore, targeting HIF-1 is expected to enhance the effect of radiation therapy. Indeed, several pre-clinical studies showed that the inhibition of intratumoral HIF-1 activity with an HIF-1 inhibitor, YC-1, significantly enhanced the therapeutic effect of radiation (Moeller *et al*, 2004). Likewise, the elimination of HIF-1-positive cells from solid tumours with a protein drug, TOP3, or with a gene therapy strategy had radiosensitising effects (Harada *et al*, 2002, 2007a; Liu *et al*, 2007). However, the inhibition of intratumoral HIF-1 activity in unsuitable timing may suppress rather than enhance the effect of radiation therapy; in that the inhibition has an anti-angiogenic effect and consequently increases the radioresistant hypoxic fraction (Yeo *et al*, 2003). To avoid this, it is important to analyse the relationship between treatment regimen and therapeutic benefit.

In this study, we performed a series of optical and real-time imaging experiments using an HIF-1-dependent reporter gene, *5HRE-ODD-luc* (Harada *et al*, 2007b), and analysed the changes in intratumoral HIF-1 activity after treatment with radiation and/or an HIF-1 inhibitor, YC-1. The imaging experiments revealed that intratumoral HIF-1 activity decreased at 6 h postirradiation, and then increased and peaked at 18–24 h postirradiation. On the other hand, YC-1 temporarily but significantly inhibited HIF-1 activity at 24 h post-injection, leading to a decrease in microvessel density and a resultant increase in tumour hypoxia. We found that the YC-1-mediated increase in the hypoxic fraction has a negative impact on the therapeutic effect of radiation when YC-1 is administered first. On the other hand, in the reverse sequence, YC-1 suppressed the postirradiation activation of HIF-1, leading to an enhancement of radiation therapy. This study emphasises the importance of imaging HIF-1 activity and determining the treatment regimen when combining radiation with an HIF-1 inhibitor.

## Materials and methods

### Cell culture and reagent

The human cervical epithelial adenocarcinoma cell line (HeLa) was purchased from American Type Culture Collection (Manassas, VA, USA). RCC4/Vector and RCC4/VHL, which are human renal cell carcinoma cell lines (RCC4) stably transfected with pcDNA3 (an empty vector) and pcDNA3-VHL (a VHL-expressing vector), respectively, were purchased from DS Pharma Biomedical (Osaka, Japan). Cells were maintained in 10% FBS-Dulbecco's modified Eagle's medium (D-MEM). For normoxic cultures, cells were incubated in a well-humidified incubator with 5% CO_2_ and 95% air at 37°C. For hypoxic cultures, cells were incubated in a Bactron Anaerobic Chamber, BACLITE-2 (O_2_ <0.02%; Sheldon Manufacturing Inc., Cornelius, OR, USA). YC-1 (Cayman Chemical Company, Ann Arbor, MI, USA) was dissolved in DMSO at a concentration of 60 mg ml^−1^.

### Isolation of stable transfectants

RCC4/Vector and RCC4/VHL were transfected with the plasmid p5HRE-ODD-luc (Harada *et al*, 2007b) by the calcium phosphate method (Chen and Okayama, 1987) to establish RCC4/Vector/5HREp-ODD-Luc and RCC4/VHL/5HREp-ODD-Luc cells, respectively, and cultured for 10 days in medium containing 400 *μ*g ml^−1^ of G418 (Nacalai Tesque, Kyoto, Japan). Antibiotic-resistant colonies showing HIF-1-dependent bioluminescence were isolated and established as clones. A representative clone was used in this study. HeLa/EFp-Luc and HeLa/5HREp-ODD-Luc cells were established as described earlier (Harada *et al*, 2005, 2007b).

### Luciferase assay for RCC4-derived stable transfectants

Cells were seeded in 24-well plates (2 × 10^4^ cells per well) and cultured under normoxic or hypoxic conditions for 18 h. The cells were washed with PBS twice and lysed with 100 *μ*l of Passive Lysis Buffer (Promega, Madison, WI, USA) for luciferase assays using Luciferase Assay Reagent (Promega) according to the manufacturer's instructions.

### Western blotting, luciferase assay, ELISA and FACS analysis *in vitro*

HeLa/5HREp-ODD-Luc cells were seeded into six-well culture dish (2 × 10^5^ per well) and treated with HIF-1*α* siRNA or scramble siRNA (Invitrogen Corp., Carlsbad, CA, USA) for 12 h. The culture medium was refreshed with 1 ml of D-MEM containing 0.1% foetal bovine serum with or without YC-1 (10 *μ*M). The cells were cultured for an additional 24 h under normoxic or hypoxic conditions, and subjected to western blotting with anti-HIF-1*α* antibody (BD Bioscience, San Diego, CA, USA) and with anti-*β*-actin antibody (BioVision Research Products, Mountain View, CA, USA) or to luciferase assay (Promega), as described earlier (Harada *et al*, 2005; Harada *et al*, 2007b). On the other hand, the normoxic or hypoxic conditional medium was subjected to human VEGF ELISA system (GE healthcare UK Ltd, Buckinghamshire, UK) to analyse the secreted VEGF level, according to the manufacturer's instructions. The normoxic or hypoxic conditional medium was given to pre-irradiated HUVEC (3 × 10^5^ cells per well in a six-well dish; 2 Gy with an X-ray irradiation (Shimadzu, Kyoto, Japan)), and the HUVEC was cultured for an additional 48 h. Apoptotic fraction (sub-G1 fraction) was analysed with FACS using propidium iodide, as described earlier (Harada *et al*, 2006).

### Tumour-bearing mice and radiation conditions

The suspensions of HeLa-derived cells (2 × 10^6^ cells in PBS) and RCC4-derived cells (2 × 10^7^ cells in PBS) were subcutaneously inoculated into the right hind leg of 6-week-old nude mice (BALB/c nu/nu mice; SHIMIZU Laboratory Supplies Co. Ltd, Kyoto, Japan) and severe combined immunodeficient (SCID) mice (C.B-17/lcr-scid/scidJcl; CLEA Japan Inc., Tokyo, Japan), respectively. The tumour xenografts were irradiated with 5 Gy of ^137^Cs *γ*-rays using a Gammacell 40 Exactor (MDS Nordion International Inc., Ontario, Canada). Local irradiation was achieved with a specific collimator (MDS Nordion International Inc.).

### Real-time imaging of luciferase activity in tumour xenografts

When the average tumour volume reached approximately 150 mm^3^, an osmotic pump (model 1007D; Alzet Osmotic Pumps, Cupertino, CA, USA) loaded with 200 *μ*l of D-luciferin (20 mg ml^−1^ in PBS; Promega) was subcutaneously transplanted into the left flank of tumour-bearing mice. The mice were treated with *γ*-ray irradiation and/or injected intraperitoneally with YC-1 (100 mg kg^−1^; see each figure legend for the treatment schedule). Optical imaging to detect luciferase bioluminescence was carried out with an IVIS-200 *in vivo* imaging device (Xenogen, Alameda, CA, USA). During the imaging, the mice were anaesthetised with 2.5% isoflurane gas in the oxygen flow (1.5 l min^−1^). Images were analysed using Living Image 2.50-Igor Pro 4.09 software (Xenogen).

### Immunohistochemical analyses

HeLa/5HREp-ODD-Luc tumour xenografts were surgically excised 90 min after an intraperitoneal injection with pimonidazole hydrochloride (Natural Pharmacia International Inc., Belmont, MA, USA; 60 mg kg^−1^). For diaminobenzidine staining of pimonidazole hydrochloride and CD31, the formalin-fixed and paraffin-embedded sections were treated with anti-pimonidazole antibody and anti-CD31 antibody respectively, as described earlier (Harada *et al*, 2007a; Liu *et al*, 2007; Zeng *et al*, 2008). For fluorescent double staining of HIF-1*α* and pimonidazole, the tumour xenografts were embedded in OCT compound and frozen at −80°C. The frozen sections were fixed in 2% paraformaldehyde and ice-cold methanol sequentially for 5 min each, blocked with blocking solution (serum-free protein block solution (Dako, Glostrup, Denmark) containing 0.1% cold water fish skin (CWFS) gelatin (Sigma-Aldrich Corp., St Louis, MO, USA)) and treated with anti-HIF-1*α* mAb (BD Bioscience) in the blocking solution. After being washed extensively with PBS, the sections were blocked with PBS containing 0.1% CWFS gelatin and treated with Alexa Fluor 546 rabbit anti-mouse IgG (Invitrogen Corp.) in the blocking solution. After further extensive washing with PBS, counter staining was conducted with DAPI (Wako Pure Chemical Industries Ltd, Osaka, Japan). The sections were next treated with FITC-conjugated anti-pimonidazole mAb (Natural Pharmacia International Inc.). For the analysis of perfusion (Hoechst 33342 distribution) and the number of functional blood vessels, tumour-bearing mice were intravenously injected with 100 *μ*l of Hoechst 33342 trihydrochloride trihydrate solution (10 mg ml^−1^; Invitrogen Corp.) 1 min before excision of each xenograft. To calculate the percentages of pimonidazole-positive and HIF-1*α*-positive cells, the positive areas were quantified using NIH Image 1.63 software (NIH, Bethesda, MD, USA) and compared with the entire tumour. The quantitative analyses were conducted in a double-blind fashion. To quantify the microvessel density, CD31-positive vessels were counted in 10 fields of five xenografts under × 40 magnifications. The quantitative analyses were conducted in a double-blind fashion.

### Growth delay assay

The tumour-bearing mice with HeLa/5HREp-ODD-Luc were intravenously injected with YC-1 (100 mg kg^−1^; see each figure legend for detailed treatment schedule) or its vehicle, and subjected to local *γ*-ray irradiation (see each figure legend for treatment schedule). The size of solid tumours was measured with calipers during and after treatments. Tumour volume was calculated as 0.5 × length × width^2^, and the tumour volume on each day was compared with the initial value to calculate the relative tumour volume.

### Ethics of animal experiments

All of our animal experiments were approved by the Animal Research Committee of Kyoto University, and the procedures were consistent with the United Kingdom Co-ordinating Committee on Cancer Research (UKCCCR) Guidelines for the welfare of animals in experimental neoplasia (second edition).

## Results

### Changes in intratumoral HIF-1 activity after ionising radiation

**Figure 1:**
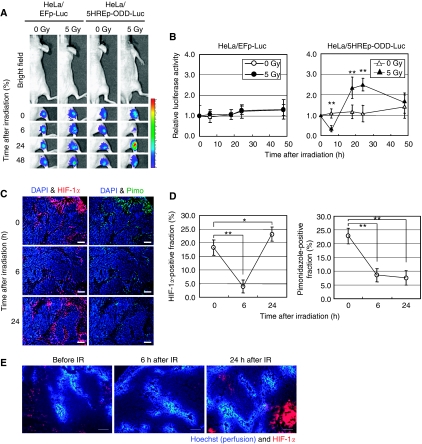
Optical imaging of intratumoral HIF-1 activity after ionising radiation. (**A**) HeLa/EFp-Luc or HeLa/5HREp-ODD-Luc xenografts were *γ*-ray irradiated at a dose of 0 or 5 Gy. Intratumoral HIF-1 activity was monitored as luciferase bioluminescence at the indicated time after irradiation. (**B**) The bioluminescent intensity from the HeLa/EFp-Luc (left) or HeLa/5HREp-ODD-Luc (right) xenograft in (**A**) was quantified. Shown in the graphs are the profiles of the relative photon count at each time point after irradiation. Results are means±s.d., *n*=6. (**C**) The HeLa/5HREp-ODD-Luc xenografts were surgically excised at the indicated time after irradiation and subjected to immunohistochemical analyses with anti-HIF-1*α* mAb (red fluorescence) or anti-Pimonidazole mAb (green fluorescence). Counter staining was conducted with DAPI (blue fluorescence). Bar=200 *μ*m. (**D**) Fractions of HIF-1*α*-positive cells (left) and pimonidazole-positive cells (right) in (**C**) were quantified. Results are means for 10 fields in five xenografts±s.d. ^*^P<0.05, ^**^P<0.01. (**E**) The HeLa/5HREp-ODD-Luc xenografts were surgically excised at the indicated time after irradiation and subjected to immunohistochemical analyses with anti-HIF-1*α* mAb (red fluorescence). A perfusion marker, Hoechst 33342 (blue fluorescence), was administrated i.v. at 1 min before each tumour excision. Bar=200 *μ*m.

To optimise the combination of radiation and inhibition of HIF-1, we first focused on the dynamics of intratumoral HIF-1 activity after ionising radiation. We performed optical imaging experiments using an HIF-1-dependent reporter gene, *5HREp-ODD-luc*, in which the 5HRE promoter enhances expression of the ODD-Luc fusion protein under hypoxic conditions (Harada *et al*, 2007b). As the 5HRE promoter depends highly on HIF-1 activity, and because the stability of the ODD-Luc protein is regulated through the same oxygen-dependent mechanism as that of HIF-1*α*, the *5HREp-ODD-luc* reporter gene is suitable for the real-time imaging of absolute HIF-1 activity (Harada *et al*, 2007b). We subcutaneously transplanted HeLa cells stably transfected with the *5HREp-ODD-luc* gene (HeLa/5HREp-ODD-Luc cells) and monitored the postirradiation dynamics of intratumoral HIF-1 activity using an optical *in vivo* imaging device (Figure 1A and B). The level of activity decreased significantly and reached a minimum at 6 h after 5 Gy of *γ*-ray irradiation (*P*<0.01). After that, HIF-1 activity increased, reached a plateau at 18–24 h postirradiation (*P*<0.01) and decreased thereafter. Immunohistochemical analyses confirmed that the level of HIF-1*α* protein at the edges of DAPI-positive viable regions correlated with that of bioluminescent intensity in the irradiated HeLa/5HREp-ODD-Luc xenografts (Figure 1C and D, left graph), indicating that the HIF-1*α* level is mainly responsible for the postirradiation HIF-1 activity in the tumour xenograft. Although the radiation-induced activation of HIF-1 and the underlying mechanisms were reported earlier (Moeller *et al*, 2004; Harada *et al*, 2009), this study is the first report to show the temporary decreases in HIF-1*α* expression and HIF-1 activity at several hours postirradiation.

### pVHL-dependent decrease in HIF-1*α* protein under radiation-induced reoxygenated conditions

As HIF-1*α* is known to be rapidly degraded under oxygen-available conditions (Jaakkola *et al*, 2001), we postulated that a radiation-induced improvement of oxygen availability (tumour reoxygenation) was involved in the temporary decrease in HIF-1*α* expression and HIF-1 activity at 6 h postirradiation. To examine this possibility, we performed an immunohistochemical analysis using a marker of hypoxia, pimonidazole (Kennedy *et al*, 1997; Figure 1C and D). Pimonidazole-positive cells showed almost the same distribution as HIF-1*α*-positive cells before irradiation (Figure 1C). The numbers of pimonidazole-positive cells predictably decreased 6 h after irradiation (Figure 1C and D, right graph), indicating that radiation-induced reoxygenation occurred in the regions. The distribution of a perfusion marker, Hoechst 33342, among the tumour xenograft was not decreased after radiation treatment, supporting the interpretation that the decrease in pimonidazole-positive cells was caused by the reoxygenation but not by the decrease in permeability of pimonidazole (Figure 1E).

**Figure 2:**
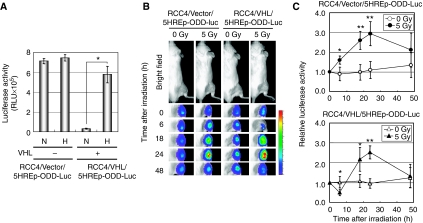
Downregulation of intratumoral HIF-1 activity at 6 h postirradiation depends on the VHL tumour suppressor gene. (**A**) RCC4/Vector/5HREp-ODD-Luc and RCC4/VHL/5HREp-ODD-Luc cells were cultured under normoxic or hypoxic conditions for 18 h and subjected to luciferase assays. Results are means±s.d., *n*=3. (**B**) RCC4/Vector/5HREp-ODD-Luc xenografts and RCC4/VHL/5HREp-ODD-Luc xenografts were irradiated at a dose of 0 or 5 Gy. The intratumoral HIF-1 activity was monitored as luciferase bioluminescence at the indicated time after irradiation. (**C**) The bioluminescent intensity from the RCC4/Vector/5HREp-ODD-Luc (upper) and RCC4/VHL/5HREp-ODD-Luc (lower) xenografts in (**B)** was quantified. Shown in the graphs are the profiles of the relative photon count at each time point after irradiation. Results are means±s.d., *n*=6. ^*^P<0.05, ^**^P<0.01.

We postulated that the temporary downregulation of HIF-1 activity resulted from the pVHL-dependent proteolysis of HIF-1*α* protein under reoxygenated conditions. To test this possibility, we took advantage of a VHL-deficient human renal cell carcinoma cell line RCC4. RCC4 cells stably transfected with the *5HREp-ODD-luc* reporter gene (RCC4/Vector/5HREp-ODD-Luc cells) showed intense bioluminescence regardless of the surrounding conditions *in vitro* (Figure 2A). On the other hand, reconstitution of the functional VHL gene (RCC4/VHL/5HREp-ODD-Luc cells) resulted in hypoxia-dependent bioluminescence (Figure 2A). We subcutaneously transplanted the cells and monitored the dynamics of intratumoral HIF-1 activity after 5 Gy of *γ*-ray irradiation (Figure 2B and C). We chose a SCID mouse as described earlier, because it was difficult to prepare RCC4 tumour xenografts in nude mice (Ogura *et al*, 2005). The HIF-1 activity in the RCC4/Vector/5HREp-ODD-Luc tumour xenograft did not decrease 6 h after irradiation but gradually increased and peaked at 24 h after irradiation. On the other hand, RCC4/VHL/5HREp-ODD-Luc xenografts showed the same pattern as HeLa/5HREp-ODD-Luc xenografts. These results clearly showed that radiation-induced reoxygenation leads to the degradation of HIF-1*α* protein through a pVHL-dependent pathway 6 h after irradiation.

### Changes in intratumoral HIF-1 activity after administration of an HIF-1 inhibitor, YC-1

**Figure 3:**
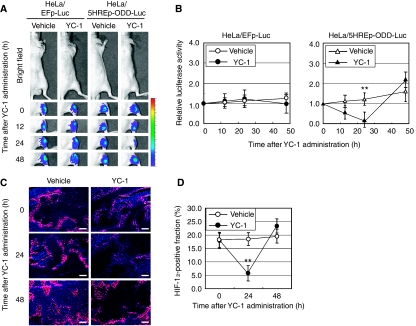
Optical imaging of intratumoral HIF-1 activity after YC-1 administration. (**A**) HeLa/EFp-Luc or HeLa/5HREp-ODD-Luc xenograft was administrated with vehicle or YC-1, and intratumoral HIF-1 activity was monitored as luciferase bioluminescence at the indicated time after the administration. (**B**) The bioluminescent intensity from the HeLa/EFp-Luc (left) or HeLa/5HREp-ODD-Luc (right) xenograft in (**A**) was quantified. Shown in the graphs are the profiles of the relative photon count at each time point after vehicle or YC-1 treatment. Results are means±s.d., *n*=6. (**C**) The HeLa/5HREp-ODD-Luc xenografts were surgically excised at the indicated time after vehicle or YC-1 administration and subjected to immunohistochemical analysis with anti-HIF-1*α* mAb (red fluorescence). Counter staining was conducted with DAPI (blue fluorescence). Bar=200 *μ*m. (**D**) Fractions of HIF-1*α*-positive cells in (**C**) were quantified. Results are means of 10 fields in five xenografts±s.d. ^**^P<0.01.

We next evaluated the effect of an HIF-1 inhibitor, YC-1, on the intratumoral HIF-1 activity with the same optical imaging experiment (Figure 3A and B). Hypoxia-inducible factor-1 activity gradually but significantly decreased and reached a minimum of 24 h after the administration of YC-1 in the HeLa/5HREp-ODD-Luc tumour xenograft (*P*<0.01). The activity then recovered to the same levels as in vehicle-injected tumours until 48 h post-injection. Immunohistochemical analysis for HIF-1*α* protein confirmed that the level of HIF-1*α* protein at the edges of DAPI-positive viable regions was dramatically decreased 24 h after the YC-1 treatment and correlated with the intensity of bioluminescence detected with the imaging device (Figure 3C and D).

### YC-1-mediated increase in tumour hypoxia suppresses the therapeutic effect of radiation

**Figure 4:**
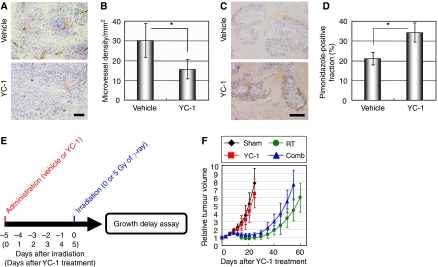
YC-1-mediated increase in tumour hypoxia suppresses the therapeutic effect of radiation. (**A**–**D**) The HeLa/5HREp-ODD-Luc xenografts were surgically excised 120 h (5 days) after the administration of vehicle or YC-1, and subjected to immunohistochemical analysis with anti-CD31 mAb (**A**) or anti-pimonidazole Ab (**C**). Bar=50 *μ*m. Microvessel density detected as CD31-positive cells in (**A**) and hypoxic cells detected as pimonidazole-positive cells (right) in (**C**) were quantified in (**B**) and (**D**), respectively. Results are means for 10 fields in five xenografts±s.d. ^*^P<0.05. (**E**) Treatment schedule of YC-1 and radiation therapy. The HeLa/5HREp-ODD-Luc tumour-bearing mice were administered with vehicle (Sham and RT groups) or YC-1 (YC-1 and Comb groups) on day 0, and subjected to 0 (Sham and YC-1 groups) or 5 Gy (RT and Comb groups) of *γ*-ray irradiation on day 5. (**F**) Growth delay assay after the treatment described in (**E**). Relative tumour volumes are calculated as the ratio of the tumour volume on each day to the corresponding volume on day 0. Results are the means for eight independent tumours±s.d.

As HIF-1 is known as a master regulator of angiogenesis, we assumed that the inhibition of intratumoral HIF-1 activity would influence the distribution and proportion of tumour hypoxia. To examine this possibility, we performed immunohistochemical analyses of CD31 (for tumour blood vessels) and pimonidazole hydrochloride (for tumour hypoxia). The YC-1-mediated HIF-1 inhibition led to a significant decrease in microvessel density (Figure 4A and B; *P*<0.05) and resultant increase in hypoxic fractions (Figure 4C and D) at 120 h (5 days) after the YC-1 treatment.

As hypoxia has been associated with the radioresistance of tumours, we speculated that the YC-1-mediated increase in hypoxia might suppress the cytotoxic effect of radiation. To examine such a possibility, we treated the HeLa/5HREp-ODD-Luc tumour xenografts with radiation 120 h (5 days) after YC-1 treatment (Figure 4E), and performed a tumour growth delay assay (Figure 4F). The single administration of YC-1 alone little influenced tumour growth compared with vehicle treatment (Figure 4F and Table 1), although it led to a significant decrease in microvessel density (Figure 4A and B). However, YC-1 treatment accelerated rather than delayed tumour growth after radiation therapy, when it was administered 120 h (5 days) before the radiation treatment (Figure 4F). Days, in which tumour volume reached two-fold of the initial volume (tumour growth doubling time), after ‘the IR treatment alone’ and ‘the combination with −5 days interval’ were ‘41.4±6.8 days’ and ‘33.8±5.4 days’, respectively (Table 1; *P*<0.05). These results indicate that the YC-1-induced increase in tumour hypoxia protects tumour cells against radiation, and HIF-1 inhibitors have a negative impact on the therapeutic effect of radiation in such an unsuitable treatment regimen.

### Suppression of postirradiation HIF-1 activation sensitises the effect of radiation therapy

**Figure 5:**
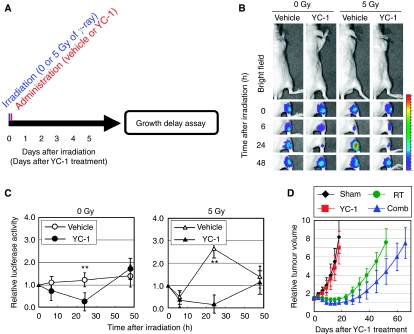
Suppression of postirradiation HIF-1 activation sensitises effect of radiation therapy. (**A**) Treatment schedule of radiation and YC-1. The HeLa/5HREp-ODD-Luc tumour-bearing mice were subjected to 0 (Sham and YC-1 groups) or 5 Gy (RT and Comb groups) of *γ*-ray irradiation on day 0, and administered with vehicle (Sham and RT groups) or YC-1 (YC-1 and Comb groups) at 1 min after the irradiation. (**B**) Intratumoral HIF-1 activity was monitored as luciferase bioluminescence at the indicated time after the radiation. (**C**) The bioluminescence detected in (**B**) was quantified. Shown in the graphs are the profiles of the relative photon count at each time point after the treatment. Results are means±s.d., *n*=8. ^**^P<0.01. (**D**) Growth delay assay after the treatment described in (**A**). Relative tumour volumes are calculated as the ratio of the tumour volume on each day to the corresponding volume on day 0. Results are the means for 8 independent tumours±s.d.

To find the optimal regimen, we next examined whether the suppression of HIF-1 activation 24 h postirradiation improves the therapeutic effect of radiation. As a maximal effect of YC-1 on HIF-1 activity was observed 24 h after its administration (Figure 3A and B), we administered YC-1 just after (at 1 min after) radiation treatment to effectively suppress the upregulation of HIF-1 activity at 24 h postirradiation (Figure 5A). Optical imaging experiments confirmed that the administration almost completely suppressed the radiation-induced upregulation of HIF-1 activity (Figure 5B and C; *P*<0.01). The HIF-1-inhibiting effect led to a delay of tumour growth compared with the radiation therapy alone (Figure 5D). Tumour growth doubling times after ‘the IR treatment alone’ and ‘the combination with +1 min interval’ were ‘26.5±5.4 days’ and ‘36.5±5.5 days’, respectively (Table 1; *P*<0.05).

**Figure 6:**
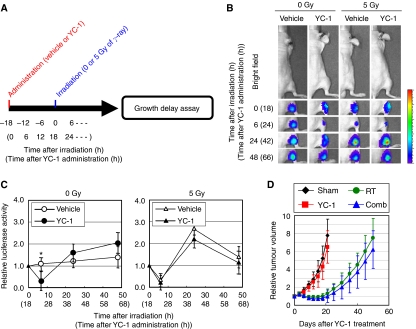
Unsuitable timing of YC-1 administration has no influence on therapeutic effect of radiation. (**A**) Treatment schedule of YC-1 and radiation therapy. The HeLa/5HREp-ODD-Luc tumour-bearing mice were administered with vehicle (Sham and RT groups) or YC-1 (YC-1 and Comb groups), and the xenografts were subjected to 0 (Sham and YC-1 groups) or 5 Gy (RT and Comb groups) of *γ*-ray irradiation 18 h later. (**B**) Intratumoral HIF-1 activity was monitored as luciferase bioluminescence at the indicated time after irradiation (**C**) The bioluminescence detected in (**B**) was quantified. Shown in the graphs are the profiles of the relative photon count at each time point after the irradiation. Results are means±s.d., *n*=8. ^*^P<0.05. (**D**) Growth delay assay after the treatment described in (**A**). Relative tumour volumes are calculated as the ratio of the tumour volume on each day to the corresponding volume on day 0. Results are the means for eight independent tumours±s.d. Time after the administration of YC-1 is given in parentheses.

To further confirm the importance of HIF-1 inhibition 24 h postirradiation, we performed the same kind of experiments with a different regimen (Figure 6). YC-1 was administered 18 h before irradiation (Figure 6A) with the expectation that the maximum HIF-1-inhibiting effect of YC-1 would come 6 h postirradiation, at which time radiation-induced reoxygenation minimised the intratumoral HIF-1 activity through a VHL-dependent mechanism (Figures 1 and 2). The optical imaging experiments confirmed that the decrease in HIF-1 activity caused by YC-1 and by the radiation overlapped each other at 6 h postirradiation, and YC-1 had no influence on the upregulation of HIF-1 activity at 24 h postirradiation with this regimen (Figure 6B and C). The administration of YC-1 had almost no impact on the therapeutic effect of radiation with this regimen (Figure 6D). Tumour growth doubling times after ‘the IR treatment alone’ and ‘the combination with −18 h interval’ were ‘27.7±6.4 days’ and ‘30.8±5.5 days’, respectively (Table 1; no significant difference between the combination and radiation alone). These results indicate that YC-1 has a radiosensitising effect only when administered to suppress the radiation-induced activation of HIF-1.

### YC-1 enhances the vascular-disrupting effect of radiation in optimal treatment regimen

**Figure 7:**
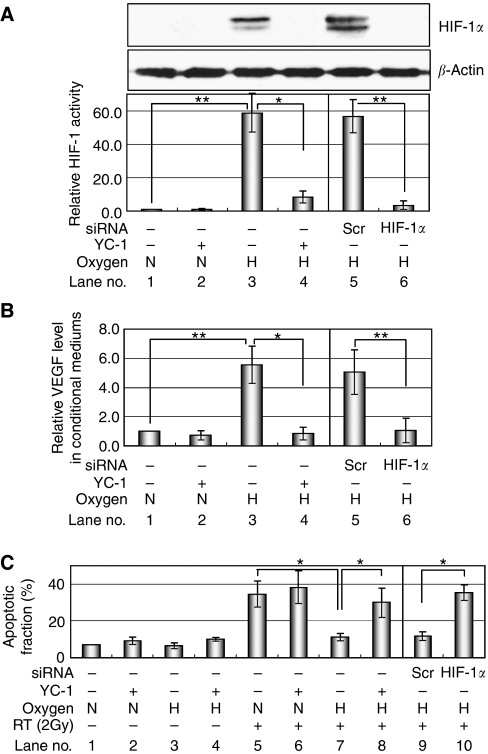
YC-1 diminishes the radioprotective effect of hypoxic conditional medium on endothelial cells *in vitro*. HeLa/5HREp-ODD-Luc cells were treated with HIF-1*α*-siRNA or Scramble (Scr)-siRNA or without both of them (−), and cultured under normoxic (N) or hypoxic (H) conditions in the presence (+) or absence (−) of YC-1. (**A**) The HeLa/5HREp-ODD-Luc lysates were subjected to western blotting for HIF-1*α* (upper) or *β*-actin (middle) and to luciferase assay (lower). (**B**) The conditional mediums of the HeLa/5HREp-ODD-Luc cells were subjected to ELISA for VEGF level. The VEGF level after each treatment was compared with that after normoxic, YC-1 (−) and siRNA (−) conditions. (**C**) HUVEC was X-ray irradiated (2 Gy) and cultured in the above conditional mediums for 48 h. The apoptotic fraction was quantified as sub-G_1_ fraction. All results are means±s.d., *n*=3. ^*^P<0.05, ^**^P<0.01.

It has been reported that HIF-1 becomes active in response to radiation-induced alteration of tumour microenvironment, induces VEGF and consequently protects endothelial cells from the cytotoxic effect of radiation (Gorski *et al*, 1999; Moeller *et al*, 2004; Zeng *et al*, 2008). On the basis of this information, we hypothesised that YC-1 has a potential to enhance the vascular-disrupting activity of radiation through the suppression of HIF-1 activity. To examine such a possibility *in vitro*, we cultured the HeLa/5HREp-ODD-Luc cells under normoxic or hypoxic conditions and obtained each conditional medium. The conditional medium was given to pre-irradiated (2 Gy of X-ray) HUVEC, and their apoptotic fraction was quantified as sub-G_1_ fraction with FACS analysis (Figure 7). The hypoxic treatment for 24 h was sufficient to induce HIF-1*α* expression (Figure 7A, upper lane 3) and HIF-1 activity (Figure 7A, lower lane 3; *P*<0.01) in the HeLa/5HREp-ODD-Luc cells. The hypoxic conditional medium contained a higher concentration of a hypoxia-dependent gene product secreted from the HeLa/5HREp-ODD-Luc cells (compare Figure 7B lane 1 with 3; *P*<0.01) and significantly protected the HUVEC from radiation-induced apoptosis compared with the normoxic counterpart (compare Figure 7C lane 5 with 7; *P*<0.05). The HIF-1*α* siRNA treatment suppressed the hypoxia-dependent expression of HIF-1*α* (compare Figure 7A upper lane 5 with 6), activation of HIF-1 (compare Figure 7A lower lane 5 with 6; *P*<0.01) and secretion of VEGF (compare Figure 7B lane 5 with 6). Consequently, the HIF-1*α* siRNA treatment suppressed the radioprotective effect of the hypoxic conditional medium (compare Figure 7C lane 9 with 10). YC-1 also suppressed the radioprotective effect of hypoxic conditional medium through the decrease in HIF-1*α* expression, HIF-1 activity and VEGF secretion (Figure 7).

**Figure 8:**
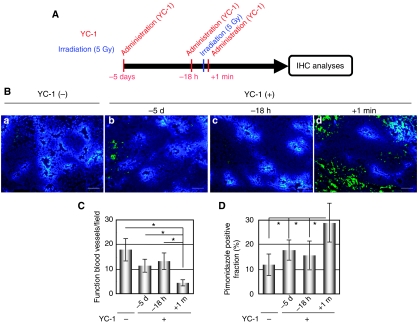
Treatment regimen affects the effect of YC-1 on the number of functional tumour blood vessels after radiation therapy. (**A**) Treatment schedule of YC-1 and radiation therapy. The HeLa/5HREp-ODD-Luc tumour-bearing mice were administered with YC-1 at the indicated time before or after *γ*-ray irradiation. (**B**) The HeLa/5HREp-ODD-Luc xenografts were treated with radiation and YC-1 in various time course as described in (**A**), surgically excised 5 days after irradiation and subjected to immunohistochemical analyses with anti-pimonidazole mAb (green fluorescence) and a perfusion marker, Hoechst 33342 (blue fluorescence). Hoechst 33342 was administrated i.v. at 1 min before each tumour excision. Bar=200 *μ*m. (**C** and **D**) The number of functional blood vessels (**C**) and fractions of pimonidazole-positive cells (**D**) in (**B**) were quantified. Results are means for 10 fields in five xenografts±s.d. ^*^P<0.05.

To examine whether such an effect of YC-1 was responsible for the best therapeutic benefit of our optimal treatment regimen in Figure 5, we performed immunohistochemical analyses for functional blood vessels and for tumour hypoxia (Figure 8) at 5 days after ionising radiation of each treatment regimen applied in Figures 4, 5 and 6 (Figure 8A). When we administered YC-1 at 1 min after radiation treatment and suppressed the postirradiation upregulation of HIF-1 activity, the number of functional microvessel was significantly decreased (Figure 8Bd and C) and the pimonidazole-positive fraction was significantly increased (Figure 8Bd and D) compared with those after the other two combination regimens. These results suggest that, in the optimal treatment regimen, YC-1 enhanced the therapeutic effect of radiation through (1) suppressing radiation-induced HIF-1*α* expression and HIF-1 activity, (2) suppressing HIF-1-dependent secretion of radioprotective protein(s) such as VEGF, (3) increasing radiosensitivity of endothelial cells and (4) decreasing microvessel density.

## Discussion

In this study, we performed optical imaging experiments and revealed that ionising radiation induces dynamic changes in intratumoral HIF-1 activity: a temporary decrease and a subsequent increase. In addition, the imaging revealed that YC-1 treatment temporarily inhibits intratumoral HIF-1 activity, leading to a decrease in tumour microvessel density and resultant increase in the tumour hypoxic fraction. On the basis of these results, we set treatment regimens combining radiation with YC-1, and compared their therapeutic efficacy. Finally, we revealed that treatment regimen determines whether an HIF-1 inhibitor enhances or inhibits the therapeutic effect of radiation and that the suppression of the postirradiation upregulation of HIF-1 activity is important for the best therapeutic benefit.

The reason why such dynamics (especially the temporary decrease) of HIF-1 activity after radiation had not been observed before seemed to be differences in experimental strategy. To date, HIF-1*α* expression in tumour xenografts has been mainly analysed immunohistochemically. However, it is impossible to follow changes in expression sequentially in individual living animals. Moreover, an alternative strategy using conventional *5HREp-GFP* or *5HREp-luc* genes was unsuitable for real-time imaging because the stability of the reporter proteins made it difficult to reflect rapid decreases in HIF-1 activity (Liu *et al*, 2005; Harada *et al*, 2007b). To overcome these problems, we applied here an HIF-1-dependent reporter gene, *5HREp-ODD-luc* (Harada *et al*, 2007b). As the stability of the ODD-Luc fusion protein is regulated through the same oxygen-dependent mechanism as that of HIF-1*α* protein, the reporter gene enabled us to perform the imaging of absolute HIF-1 activity in real time. Therefore, we could show here that intratumoral HIF-1 activity initially decreases several hours after irradiation and then increases thereafter. Applying the imaging system to a VHL-deficient RCC4 cell line, we could identify the PHD-VHL pathway as critical to the temporary decrease in HIF-1 activity after radiation.

Knockdown of HIF-1*α* protein almost completely inhibited the expression of HIF-1*α* under hypoxic conditions (Figure 7A, upper lane 6), resulting in about 90% suppression of the *5HREp-ODD-luc* reporter gene activity (Figure 7A, lower lane 6). Moreover, YC-1 led to the same results as the HIF-1*α* knockdown (Figure 7A, lane 4). These results confirmed the specificity of the *5HREp-ODD-luc* reporter gene and YC-1 to HIF-1.

Immunohistochemical analysis using a marker of hypoxia revealed that the radiation-induced reoxygenation continued until at least 24 h postirradiation. In addition, we have confirmed that radiation efficiently induced apoptosis of normoxic tumour cells surrounding tumour blood vessels (data not shown). Moreover, Bristow and Hill (2008) reported that there is an increase in residual DNA double-strand breaks within normoxic tumour regions (indicated by an increased 53BP1 nuclear foci). These results are consistent with a well-known model underlying postirradiation reoxygenation that radiation-induced damage of tumour cells surrounding tumour blood vessels leads to the decrease in oxygen consumption in these regions and an increase in oxygen availability in hypoxic tumour cells.

Our optical imaging experiments and immunohistochemical analyses revealed that HIF-1*α* protein accumulated even under radiation-induced reoxygenated conditions. These results are consistent with an earlier report that radiation-induced reoxygenation leads to the formation of ROS and inhibits PHD activity, resulting in the stabilisation and accumulation of HIF-1*α* protein (Moeller *et al*, 2004). In addition, we recently identified glucose- and Akt/mTOR-dependent translation of HIF-1*α* protein as an additional crucial mechanism in the upregulation (Harada *et al*, 2009). The relationship between the ROS-related stabilisation and the glucose- and Akt/mTOR-related translation of HIF-1*α* had not yet been elucidated; however, we recently reported that the ROS-mediated stabilisation of HIF-1*α* protein alone could not be fully responsible for the activation of HIF-1 without newly translated HIF-1*α*, and both the mechanisms function coordinately in the induction of HIF-1 activity after irradiation *in vivo*.

Our optical imaging experiments visualised the target specificity of an HIF-1 inhibitor, YC-1: treatment with YC-1 led to a decrease in microvessel density in solid tumours. Such an activity resulted in an increase in tumour hypoxia 5 days after the administration of YC-1 in our experimental setting. The YC-1-mediated increase in tumour hypoxia had a negative impact on the effect of radiation therapy and accelerated tumour growth after the therapy, when it was administered 5 days before the radiation. This result is reasonable in terms of the ‘oxygen effect theory’ (Brown and Wilson, 2004), although HIF-1 targeting has been believed to be a promising strategy for enhancing the effect of radiation. In addition to the theory, suppression of ATP metabolism, proliferation and p53 activation by the inactivation of HIF-1 in this time course might have been responsible for the negative impact of YC-1 on the effect of radiation therapy, as has been reported earlier (Moeller *et al*, 2005). On the other hand, when administered at a time suitable to suppress the postirradiation upregulation of HIF-1 activity, YC-1 dramatically enhanced the therapeutic effect of radiation and significantly delayed tumour growth compared with radiation therapy alone. Our *in vitro* and *in vivo* experiments revealed the mechanism responsible for the radioenhancing effect of an HIF-1 inhibitor through the suppression of radiation-induced HIF-1*α* expression and HIF-1 activity, decrease in HIF-1-dependent secretion of radioprotective protein(s) such as VEGF, increase in radiosensitivity of endothelial cells and decrease in microvessel density. This interpretation is well consistent with the earlier reports (Gorski *et al*, 1999; Moeller *et al*, 2004; Zeng *et al*, 2008).

Although we have shown here the importance of suppressing intratumoral HIF-1 activity after radiation therapy, we should pay attention to the other possibility. It was reported earlier that YC-1 suppresses HIF-1*α* accumulation through the inhibition of various signalling pathways, including PI3K/Akt/mTOR/4E-BP and NF-kappaB (Sun *et al*, 2007). In addition, YC-1 is known to have an interesting property arresting cell cycle at S phase and inducing apoptosis in hypoxic cells (Yeo *et al*, 2006). The difference in the therapeutic effect of three treatment regimens therefore may depend on such YC-1 properties as well as on the ‘oxygen effect theory’.

Considering the vascular-disrupting effect of radiation therapy, we hypothesised that recurrent tumour may be hypoxic and HIF-1 active. If it is true, postirradiation YC-1 treatment should enhance the therapeutic effect of radiation. However, in our experimental setting, there was no significant difference in the intratumoral HIF-1 activity between ‘irradiated group’ and ‘non-irradiated group’ in the latter time points (48, 72, 96 and 120 h postirradiation), although HIF-1 activity was upregulated at 24 h postirradiation (data not shown). Moreover, YC-1 treatment at 48 h postirradiation had no radioenhancing effect (data not shown). These results are not consistent with the above-mentioned speculations; however, they support our opinion that the suppression of postirradiation upregulation of HIF-1 activity is most important for the best therapeutic benefit.

This study raises the important possibility that other strategies that lead to an increase in tumour hypoxia might have the same negative influence on the therapeutic effect of radiation as YC-1. This might be true of anti-angiogenic agents and anti-vascular agents as well as other HIF-1-targeting therapeutics. Although it has been confirmed that anti-angiogenic agents enhance the effect of radiation therapy in various basic and clinical studies, the benefit might further be increased by optimising the regimen using an HIF-1 imaging strategy. As HIF-1 activity is not fully dependent on hypoxia, HIF-1-positive cells do not necessarily overlap with absolute hypoxic regions. From this point of view, it is necessary to develop a strategy to assess intratumoral HIF-1 activity in real time, in addition to the development of methods to assess absolute hypoxia.

## Figures and Tables

**Table 1:** Statistical analysis of TGDT

	**Figure 4 (−5 days)**	**Figure 5 (+1 min)**	**Figure 6 (−18 h)**
Sham	10.0±3.1	4.8±1.9	8.0±3.9
YC-1	12.2±3.6^NS1^	5.1±2.1^NS1^	9.3±3.4^NS1^
RT	41.4±6.8	26.5±5.4	27.7±6.4
Combination	33.8±5.4^*^	36.5±5.5^*^	30.8±5.5^NS2^

TGDT=tumour growth doubling time.

NS1, not significant *vs* Sham group; NS2, not significant *vs* RT group.

TGDT was calculated as the mean of the days in which relative tumour volume of each tumour reached two-fold of the volume on day 0. Data were based on the results of the growth delay assays in Figures 4, 5 and 6. Results are the mean of the days after YC-1 treatment±s.d. (*n*=8).

^*^P<0.05 *vs* RT group.
